# Safety of total hip arthroplasty for femoral neck fractures using the direct anterior approach: a retrospective observational study in 86 elderly patients

**DOI:** 10.1186/s13037-016-0100-2

**Published:** 2016-05-06

**Authors:** Grégoire Thürig, Jürgen Wilfried Schmitt, Ksenija Slankamenac, Clément M. L. Werner

**Affiliations:** Departement of Traumatology, University Hospital Zurich, Rämistrasse 100, 8091 Zürich, Switzerland; Departement of Visceral and Transplant Surgery, University Hospital Zurich, Rämistrasse 100, 8091 Zürich, Switzerland

## Abstract

**Background:**

The femoral neck fracture is one of the most common fractures in the elderly. A variety of methods and approaches are used to treat it. Total hip arthroplasty is a preferred approach in independent, mobile, elderly patients, given its more favorable long-term outcome.

Our hypothesis is that the direct anterior approach in geriatric trauma patients has a lower dislocation-rate with the advantage of early recovery due to a muscle sparing approach and therefore early possible full weight-bearing.

**Methods:**

Patients were retrospectively sought who suffered a femoral neck fracture from 2008 to 2013. All patients were treated through a direct anterior approach and using the same brand of implants. Medical history, standardized physical exam, conventional pelvic plain and axial hip x-rays, Harris Hip Score, Merle D'Aubigné and Postel and SF-36 were assessed.

**Results:**

Eighty-six patients were included in the study with a mean age of seventy-five years. The mortality rate was 16.7 %. Complications were encountered in nineteen patients (22.0 %) who needed operative revision and one postoperative complication (1.2 %) which could be handled conservatively. There were five intraoperative complications (5.8 %), two dislocations (2.3 %), one aseptic loosening in a non-cemented stem (1.2 %), six periprosthetic fractures in non-cemented stems (6.9 %), one displacement of a non-cemented cup (1.2 %), two early infections (2.3 %) and three hematomas (3.5 %) recorded.

**Conclusions:**

Although the direct anterior approach is associated with a rather long learning curve we have found it to preserve the soft-tissues with no injury to abductors. It therefore shows an early advantage in elderly patients in terms of early recovery and therefore early possible full weight-bearing. Fracture treatment with dual mobility cups might lead to lower dislocation rates, but are associated with higher costs. Due to higher complication rates in non-cemented versus cemented shafts, we have changed our practice towards favoring cemented femoral stems in patients with suspected or manifest osteoporosis.

## Background

The femoral neck fracture is one of the most common fractures in the elderly [1]. Younger patients are also frequently affected due to accidents. In terms of surgical treatment, there are a variety of methods for hemiarthroplasty and total arthroplasty. In ambulatory patients, total hip replacement is the preferred treatment, given its more favorable long-term outcome [2–10]. In terms of approach – direct anterior approach [11–16], anterolateral approach [17, 18], lateral approach [19, 20], posterolateral approach [21], posterior approach [17, 22, 23], 2-incision approach [17] – the methods are often chosen independently of scientific studies and often depend on the local economic and social conditions and/or preferences of surgeons.

The aim of this study is to review the outcome after treatment with a direct anterior approach minimal invasive total hip arthroplasty compared with the outcomes of different methods of surgical treatment of femoral neck fractures described in the literature. Our hypothesis is that the direct anterior approach in geriatric trauma patients has a lower dislocation-rate with the advantage of early recovery due to a muscle sparing approach and therefore early possible full weight-bearing.

## Methods

The retrospective study was approved by the Ethical Review Board (Ref. No.: 2011-0320).

Patients were retrospectively sought who suffered a femoral neck fracture and were all operated on by two senior surgeons in the period from January 2008 to April 2013. The patients were active prior to the accident, which means they easily walked and had an active social life. Patients with prior operations on the concerning fracture side, lateral displaced neck fractures or pathological fractures, were excluded.

All patients were treated by total hip arthroplasty through a direct anterior approach under general anesthesia and were placed in a dorsal decubitus position on an extension table. Antibiotics were applied as prophylaxis before operation in all cases.

The incision was made proximal in the intermuscular plane in-between the m. tensor fasciae latae and the m. sartorius (super fascial layer) and m. rectus femoris (deep layer). The ascending branch of the arteria circumflexa femoris lateralis was ligated and dissected. The reflected tendon of the rectus femoris was released. The anterolateral joint capsule was incised in the shape of a “V”. The pretrochanteric tubercle was then visible and the osteotomy of the femoral neck was done with an oscillating saw. The femoral head was extracted with a corkscrew. Now the acetabulum was fully exposed and the ligament capitis femoris was cauterized. The acetabular component was positioned after step-wise reaming. Exposure on the proximal femur was done by extension and external rotation after capsular release of the ischiofemoral and pubofemoral ligaments. The rasping and insertion of the shaft was done with precaution. If the temporary shaft was tightly positioned and stable, a cementless shaft was used, otherwise a medullary plug and cemented shaft was put in. In general a cemented shaft was chosen in patients showing poor bone quality or osteoporosis. Intraoperative control of cup and shaft positioning was always performed by fluoroscopy and adjusted as needed to assure optimal positioning. Closure was done by suturing the tensor fascia and the skin. A subcutaneus drain was only placed if acetylsalicylate was taken as medication.

The drainage was removed 24 h after surgery. Antibiotic prophylaxes lasted 24 h. The patients were instructed to mobilize with full weight bearing from the first postoperative day. Conventional pelvic plain and axial hip x-rays were taken after mobilization. A thrombosis prophylaxis was applied for six weeks. Follow-up controls were after six weeks, three months, six months and at least one year after surgery.

All implants were Medacta products: all cups were Versafit and all stems were Quadra. PALACOS® bone cements were used if needed.

At time of the agreed follow-up medical history was taken and standardized physical examination was performed. Conventional pelvic plain and axial hip x-rays were taken to evaluate positioning and loosening of the implants and heterotopic ossification. No MRI has been done to investigate soft-tissue damage. Additionally, three standard questionnaires (Harris hip score, Merle D'Aubigné and Postel, SF-36) and parameters such as pain, quality of life, happiness and mobility were assessed.

Secondary outcome parameters were also recorded: intraoperative and postoperative complications (major ones which needed operative revision and minor ones which could be handled conservatively).

Statistical analyses were performed using the statistical program STATA (version 12, Stata Corp., College Station, Texas). We expressed distribution of variables using means and standard deviation (SD) for normally distributed data, and medians and interquartile ranges for non-normally distributed data. We tested data for normality with the Kolmogorow-Smirnow test and performed quantile-quantile plots of dependent variables [24].

## Results

Eighty-six patients were included in the study (Table 1). The mean age was seventy-five years (68 – 81). Sixty-two were female (72.1 %) and twenty-four male (27.9 %).

**Table 1:** General information about patients

	# of patients *n* = 86
Age (years)	75 (68 – 81)
Sex (male/female)	24 / 62 (27.9 %/72.1 %)
Side	
- Right	49 (57 %)
- Left	37 (43 %)
Death (%)	24 (27.9 %)
Loss of follow up (%)	16 (18.6 %)
No participation (%)	8 (9.3 %)
Controlled	32 (37.2 %)
Interviewed	6 (7.0 %)
Follow up (months)	*n* = 65
	20 (12 – 30)
Mortality rate	16'744/100'000
Patients who needed another operation (%)	11 (12.8 %)
Pre-operative Hb (g/L)	127 (114 – 135)

All results were reported as median (25th-75th percentile)

Twenty-four (27.9 %) had already died at time of follow-up. The mortality rate was 16.7 %. Sixteen (18.7 %) couldn’t be reached despite repeated attempts to contact both by letter and telephone. Eight (9.3 %) didn’t want to take part in the study due to medical comorbidities (*n* = 6) or personal circumstances (*n* = 2). We had a total of eighty-six (*n* = 86) with a follow-up time between zero and sixty-one months; however they were still represented in terms of complications. There were *n* = 65 patients with a mean follow-up of twenty months (12 – 30). *n* = 32 were clinically controlled and filled up the three standard questionnaires, *n* = 6 were only interviewed plus filled out SF36-score.

A total of nineteen complications which needed operative revision and one postoperative complication which could be handled conservatively were encountered (Table 2):

**Table 2:** Complications

Kind of complications	*n* = 20 (23.2 %)	Need for reoperation *n* = 19 (22.0 %)	Way the complication was handled
- Femoral shaft fracture	4 (4.6 %)	4 (4.6 %)	cerclage, cemented stem
- Calcar fracture	1 (1.2 %)	1 (1.2 %)	change of approach
- Dislocation	2 (2.3 %)	2 (2.3 %)	closed reduction
- Sintering	1 (1.2 %)	1 (1.2 %)	larger neck size
- Periprosthetic fracture	6 (6.9 %)	6 (6.9 %)	plate osteosynthesis, cerclage, cemented stem, augmentation, total exchange
- Cup displacement	1 (1.2 %)	1 (1.2 %)	cemented cup
- Infection	2 (2.3 %)	2 (2.3 %)	exchange of head & inlay
- Hematoma	3 (3.5 %)	2 (2.3 %)	lavage, punction
- Thrombosis (%)	0 (0.0 %)	0 (0.0 %)	-
- Pulmonary embolism (%)	0 (0.0 %)	0 (0.0 %)	-

All results were reported as median (25th-75th percentile)

None of the patients died during the operation. One patient died on the first postoperative day from circulatory failure. Another one died six days after the operation due to a complication from a secondary independent intervention for vascular-reconstruction.

There were five intraoperative fractures (5.8 %). Four non-displaced proximal femoral shaft fractures occurred while inserting the stem and made intraoperative cerclages necessary. Despite cerclage the prosthesis had no firm grip in two cases, so a cemented shaft had to be used instead. Pertrochanteric fracture occurred while inserting the stem and needed an intraoperative change to a lateral approach and plate osteosynthesis. All patients were female.

Two dislocations (2.3 %) occurred (twenty-five days and eighteen months after surgery). None of the stems were cemented or showed sign of sintering. One had a head-size of 28 and the other 32. Both of them could be treated by closed reduction and conservative management. The reason to be submitted to the hospital was because of pain after an uncontrolled movement.

The femoral component of one non-cemented arthroplasty (1.2 %) sintered in less than six weeks and needed revision due to leg length discrepancy and consecutive weakness of the abductor muscles. Despite sintering the shaft was still in a good positioning so that only the head needed to be replaced by a larger neck size.

In six cases (6.9 %) of all cementless shafts a periprosthetic fracture occurred either observed early after surgery (seven, twenty-three, twenty-nine or fifty-five days) or after several months (fourteen weeks or six months). No fractures were observed in cemented stems. Two cases needed revision by plate osteosynthesis and cerclages keeping the stem in place, two needed to be replaced by cemented shaft with additional cerclages, one case needed a total exchange of the arthroplasty with additional cerclages and autograft strut augmentation and one case needed to be replaced by cemented shaft. Two of them had signs of loosening prior to fracture. The reason for the fracture was in three cases a fall, in two cases an aseptic loosening and in one case idiopathic.

In one case (1.2 %) a displacement of the cup had to be revised fourteen days after surgery using a cemented cup.

Two patients (2.3 %) had an early infection which could both be handled by early revision by long-term antibiotics. The heads and inlays were changed along with complete capsulotomy, debridement and lavage.

Three hematomas occurred within twenty days after surgery. Two of them (2.3 %) made a revision operation necessary while one (1.2 %) could be treated by CT-guided puncture. The microbiology showed a negative result in all patients.

Treatment with low molecular weight heparin has been administered in all patients for 6 weeks. Neither a deep vein thrombosis nor a pulmonary embolism occurred after total hip arthroplasty.

Mean ranges of motion were 120° for flexion, 20° for internal rotation, 40° for external rotation, 40° for abduction and 20° for adduction (Table 3).

**Table 3:** Range of motion

	*n* = 86
Flexion	120° (110° – 130°)
Internal rotation	20° (15° – 30°)
External rotation	40° (30° – 45°)
Abduction	40° (32.5° – 50°)
Adduction	20° (±7.5°)

All results were reported as median (25th-75th percentile)

The pain VAS-score was 0, the Harris Hip Score was 94, the Merle D’Aubigné was 11, the SF-36 physical health summary score was 41.3 and mental health summary score was 51.9 and the ASA-Score showed 5.9 % grade I, 43.0 % grade II, 46.5 % grade II and 4.6 % grade IV (Tables 4 and 5).

**Table 4:** Scores

Visual analog scale (*n* = 35)	0 (0 – 4)
Harris Hip Score (*n* = 32)	94 (88 – 99.5)
Merle d’Aubigné (*n* = 32)	11 (10.5 – 12)
ASA-Score (*n* = 86)	
- Grade I	5.9 %
- Grade II	43.0 %
- Grade III	46.5 %
- Grade IV	4.6 %

All results were reported as median (25th-75th percentile)

**Table 5:** Scores

SF-36 scale score (*n* = 36)	# of patients *n* = 36 scale score	# of patients *n* = 36 norm-based scale score
Physical functioning	57.5 (29.6)	39.3 (12.4)
Physical Role functioning	54.2 (28.3)	43.3 (12.6)
Bodily Pain	65.0 (28.3)	47.7 (12.1)
General health perception	58.1 (18.6)	44.4 (8.7)
Vitality	58.0 (17.7)	50.5 (8.4)
Social role functioning	83.6 (18.6)	49.1 (9.5)
Emotional role functioning	69.4 (41.7)	45.7 (13.2)
Mental health	72.8 (19.0)	48.6 (10.8)
	# of patients *n* = 36 summary score	
Physical health summary score	41.3 (10.9)	
Mental health summary score	51.9 (10.9)	

All results were reported as mean (standard deviation)

The cup inclination angle was 44° (39° – 49°). Using the Brooker classification of ectopic ossification [25] twenty-four (27.9 %) were diagnosed with heterotopic ossifications (Table 6).

**Table 6:** Radiology

	*n* = 86
Cup inclination	44° (39° – 49°)
Heterotopic ossification	
Brooker’s classification [33]	
- Grade 0	62 (72.1 %)
- Grade I	19 (22.1 %)
- Grade II	4 (4.6 %)
- Grade III	1 (1.2 %)
- Grade IV	0 (0.0 %)

In eight cases (9.3 %) a local sensory disturbance in the region of the scar was described, but perceived as trifling. The average leg length discrepancy was 0.16 cm (±0.44) and was always under 2 cm.

Mean operation time was 90 min, mean blood loss during the operation was 500 ml, needed blood-transfusion was *n* = 25, number of patients who needed additional surgery due to multiple injuries was *n* = 11 and mean hospitalization time was 11 days (Table 7).

**Table 7:** General information

	*n* = 86
Blood loss (mL)	500 (325 – 600)
Postoperative transfusion (%)	25 (29.1 %)
- # of EC	0 (0 – 2)
- min - max	0 – 6
Operation time (minutes)	90 (70 – 110)
Antibiotics (%)	86 (100 %)
- Duration of antibiotics (hrs.)	24 hrs
Length of hospital stay (days)	11 (10 -14)
Rehabilitation (%)	48 (55.8 %)
Length of rehabilitation (days)	21 (0 – 21)
Other operations (%)	11 (12.8 %)

All results were reported as median (25th-75th percentile)

## Discussion

There are several studies in the literature regarding the advantage of the anterior approach of total hip arthroplasty for primary osteoarthritis, avascular necrosis of the femoral head or dysplasia [13, 15, 26–30]. But no studies described the outcome exclusively for medial femoral neck fractures in the elderly treated by this procedure.

Our intraoperative complications using the direct anterior approach were slightly higher than described in the literature (1-5.4 %) [13, 31, 32]. However this is due to the fact that – as opposed to the results in the literature – we were not dealing with elective patients. The cause is more likely explained due to prior traumatic event, metabolic bone disorder, and that they occurred only in females.

In our study, dislocation occurred in two cases (2.3 %). This is less than what has been described in the studies 4-17.9 % [3, 10, 33–37] and 7.6-17.2 % in the meta-analyzes [5–7].

When compared to other investigators using a minimally invasive approach for femoral neck fractures, some authors seem to have a lower dislocation rate, but had either a shorter time of follow-up, did not specify which approach was used or had a younger control group [2, 38, 39].

Jacquot et al. [23] described for a modified postero-postero-lateral approach zero dislocation out of one-hundred and two treated femoral neck fractures after a six weeks follow-up. It is not known if after a longer follow-up period dislocations could occur. In his study dual mobility cups have been used as primary implants (see Table 8 for an overview related to the different approaches).

**Table 8:** Comparison

	Approach	Age (years)	Patients	Follow-up (months)	Dislocation (%)	HHS	SF-36 Mean Physical/Mental
Baker [33]	Lateral	74	40	36	7.5		40.53/52
Blomfeldt [38]	Anterolateral	81	60	12	0	87.2	
Dorr [34]	Posterior	69	39	48	17.9		
Keating [35]	Posterior/Lateral	75	69	24	4		
Jaquot [23]	Postero-posterolateral	79	102	1.5	0		
Macaulay [10]	Posterolat/Anterolateral	82	17	24	5.8	84.2	40.2/55.7
Park [3]	2-incision	72	44	24	4.5	88.3	
Mouzopoulos [39]	NS	73	43	48	0	83.7	
Skinner [36]	Posterolateral	81	89	12	15.7		
Van den Bekerom [37]	Posterolateral/(Antero)lateral	82	115	60	7	75.2	
Wani [2]	Posterolateral	65	50	18	0	93.7	
Burgers [7]	Meta-Analysis				8.9	81	
Yu & Wang [6]	Meta-Analysis				7.6		
Zi-Sheng [5]	Meta-Analysis	69-81	561	12-156	17.2		
OUR	Anterior	75	86	20	2.3	94	41.3/51.9

Maybe the dual mobility cup implant might be safer in terms of dislocation rate, but is associated with higher costs. In our department this type of implant is used only in revision-arthroplasty for that reason.

The cause for sintering and postoperative periprosthetic fractures (Table 2) was multifactorial. Poor bone quality due to metabolic bone disease is as well a known risk factor [40]. Marsland [41] described that 70 % had, prior to a periprosthetic fracture, signs of stem loosening. Most of our patients suffered a low-energy trauma which is described to be the leading cause for postoperative periprosthetic fractures [42]. The overall incidence is about 4.1 % and is higher for cementless shafts [31]. For those who suffered a femoral neck fracture, the risk to suffer a periprosthetic fracture is higher as well [40, 43, 44]. Measures like prescription of supplements and routine follow-up could have a preventive effect [41].

Superficial infection rate (2.3 %) without proof of deep infection is comparable to others. There is no publication describing an infection rate for the direct anterior approach in trauma patients. Published infection rates for other approaches are 0-8 % [2, 45] and a meta-analyses described an average infection rate of 3.8 % [6] (Table 9).

**Table 9:** Comparison

	Approach	Infection (%)	Thrombosis (%)	Pulmonary embolism (%)	Operation time (min)	Mor-tality %	Hospital stay (days)
Baker [33]	Lateral	7.5					
Blomfeldt [38]	Anterolateral	1.6			102	5	
Dorr [34]	Posterior					18	
Keating [35]	Posterior/Lateral	4	6	1	73.7		
Jaquot [23]	Postero-posterolateral	0	0	0	100		6.8
Macaulay [10]	Posterolat/Anterolateral	0		5.8	89.1	23.5	7.7
Park [3]	2-incision	0	0	0	70		15.1
Mouzopoulos [39]	NS						8.3
Skinner [36]	Posterolateral						
Van den Bekerom [37]	Posterolateral/(Antero)lateral					54	18.4
Wani [2]	Posterolateral	8		2	100		11.9
Burgers [7]	Meta-Analysis					13.5	
Yu and Wang [6]	Meta-Analysis						
Zi-Sheng [5]	Meta-Analysis	3.8					
OUR	Anterior	2.3	0	0	90	16.7	11

No pulmonary embolisms or deep vein thrombosis were observed in this study. In the literature, a rate of pulmonary embolism of 0-5.8 % [2, 3, 10, 23, 35] and a deep vein thrombosis rate of 0-6 % [3, 23, 35] are described (Table 9). We guess this good result is due to early mobilization with allowed full immediate weight-bearing combined with anticoagulant-treatment in prophylactic dosage.

Patients reported a low pain VAS score. We haven’t found another study using this type of score.

The mean achieved Harris Hip Score was 94 and Merle D’Aubigné score 11. This Harris Hip Score seems to be slightly better than other studies which reported a score of 75.2-93.7 [2, 3, 10, 37–39] or meta-analysis which described an average 81 [7] (Table 8). We think that this results from a muscle-sparing procedure and an early full weight-bearing.

The measured patient’s functional and mental health with SF-36 showed similar results as in Baker’s [33] or Macaulay’s [10] studies.

Heterotopic ossification can lead to pain, muscle-insufficiency and restriction in range of motion. None of the heterotopic ossification had major restriction in range of motion. We did not use any prophylactic measures and had a better outcome than in most of the major studies regarding heterotopic ossification which reported different results.

Eggli [46] had 29.2 % with grade I, 10.5 % with grade II an 4.2 % with grade III. He figured out that a lateral or anterolateral approach is associated with a higher rate of heterotopic ossification. Neal [47] described 43 % of heterotopic ossification (9 % severe) and concluded that heterotopic ossification are more frequent in total hip arthroplasty than believed and is a major cause of motion disability. Pavlou [48], in his retrospective study, showed an overall of 24 % and found out that male sex, lateral approach, and total cemented implants are significantly associated with heterotopic ossification. Chémaly [49] reports an overall incidence of heterotopic ossification of 38 % (*n* = 15), with nine severe grade III cases regarding reconstruction of acetabular fracture through total hip replacement.

Compared to other approaches without prophylactic measures our findings showed less heterotopic ossification and thus a clear advantage. We assume that a less invasive approach leads to less traumatized tissue and, in long term, to less heterotopic ossification. Bergin et al. [50] and Meneghini et al. [51] confirmed an intermuscular approach minimizes trauma to the soft tissues. Our results need to be confirmed in a study with a larger cohort. Also a better result taking prophylactic measures need to be confirmed in a study. Until then, we won’t change our treatment algorithm.

The intraoperative blood loss (Table 6) is comparable to other approaches which showed an average of 385 ml – 921 ml [2, 3, 23]. This might prove that the amount of blood loss is not directly related to one approach.

Hospital stay (Table 6) included waiting time until discharge to a rehabilitation facility and is handled differently from country to country. Also, some of our patients had multiple injuries and required longer hospital stays for different reasons. Other studies treating patients with femoral neck fractures described 6.8 days to 18.4 days [2, 3, 10, 23, 37, 39] . The heterogeneous group makes it difficult to compare whether one approach leads to shorter hospitalization.

The limitations of the study include the number of patients lost to follow-up, the variety in follow-up periods and the lack of a control group. The strength of this study is that it focuses on one pathology, continuously treated with the same approach and using the same implants.

## Conclusion

Although the direct anterior approach is associated with a rather long learning curve [52] we have found it to preserve the soft-tissues and no injury to abductors. It therefor shows an early advantage in elderly patients in terms of early recovery and therefore early possible full weight-bearing. Fracture treatment with dual mobility cups might lead to a lower dislocation rates, but are associated with higher costs. Due to higher complication rate in non-cemented versus cemented shafts we have changed our practice towards favoring cemented femoral stems in patients with suspected or manifest osteoporosis.
